# Molecular dynamics simulations of mechanical failure in polymorphic arrangements of amyloid fibrils containing structural defects

**DOI:** 10.3762/bjnano.4.50

**Published:** 2013-07-04

**Authors:** Hlengisizwe Ndlovu, Alison E Ashcroft, Sheena E Radford, Sarah A Harris

**Affiliations:** 1 School of Physics and Astronomy, University of Leeds, Leeds LS2 9JT, UK; 2 Astbury Centre for Structural Molecular Biology, University of Leeds, Leeds, LS2 9JT, UK; 3 School of Molecular and Cellular Biology, University of Leeds, Leeds LS2 9JT, UK

**Keywords:** amyloid, fibril fragmentation, steered molecular dynamics (SMD), structural defects

## Abstract

We examine how the different steric packing arrangements found in amyloid fibril polymorphs can modulate their mechanical properties using steered molecular dynamics simulations. Our calculations demonstrate that for fibrils containing structural defects, their ability to resist force in a particular direction can be dominated by both the number and molecular details of the defects that are present. The simulations thereby suggest a hierarchy of factors that govern the mechanical resilience of fibrils, and illustrate the general principles that must be considered when quantifying the mechanical properties of amyloid fibres containing defects.

## Introduction

Amyloid fibrils are biomaterials that are commonly associated with human disease [1]. Over recent years, however, properties such as self-assembly and robustness have increasingly made them attractive candidates for use in nanotechnological applications [2–3] that range from conducting nanowires [4], to drug-delivery devices [5], structural scaffolds [6–7] and functionalised hydrogels [8]. A central theme in each of these distinct potential applications is an ability to control and modulate a desired property of the fibril aggregates. The requirements for the mechanical robustness of ideal, long, conducting nanowires, for instance, is that they not be prone to fragmentation, whereas a drug-delivery device needs to be sufficiently robust to carry its cargo to the target site, but then be able to release it in response to an external signal. The ability to control the length of fibrils by using simple changes in growth and storage conditions has been successfully demonstrated for bovine insulin fibrils [9]. Before we can design fibrils with bespoke material properties, however, we first need to understand how the arrangement of the individual β-sheets modulates their mechanical behaviour.

Amyloid fibrils, like many crystalline materials, exhibit polymorphism. The predominant polymorph obtained by protein or peptide self-assembly depends on the environmental growth conditions such as pH, temperature, salt concentration and mechanical agitation [10]. Since amyloid polymorphs have been observed with drastically different morphologies [11] and chemical properties [12], it is important to develop an understanding of how the polymorphic form influences the mechanical properties of fibrils. A wealth of information on the material properties of amyloid is already available from extensive pathological and biological studies that focus on the diseases aspect of amyloid, as summarised in a recent review [13]. The mechanical properties of amyloid materials have also been characterised through various biophysical techniques [14]. These include the use of atomic force microscopy (AFM), and in particular, AFM nanoindentation methods to deduce the elastic properties of amyloid [15–19]. Computer simulations that characterise the mechanical properties of amyloid fibrils have proved useful in both verifying and expanding on the experimental work. Such computational studies have for instance, reported elastic properties of Aβ fibrils comparable to experimental values [20], investigated fibril failure under tensile loading [21], revealed that geometrical confinement of β-sheets in spider silk leads to mechanical enhancement [22], and highlighted the role played by the peptide sequence on the mechanical resistance of amylin-derived fibrils [23].

**Figure 1:**
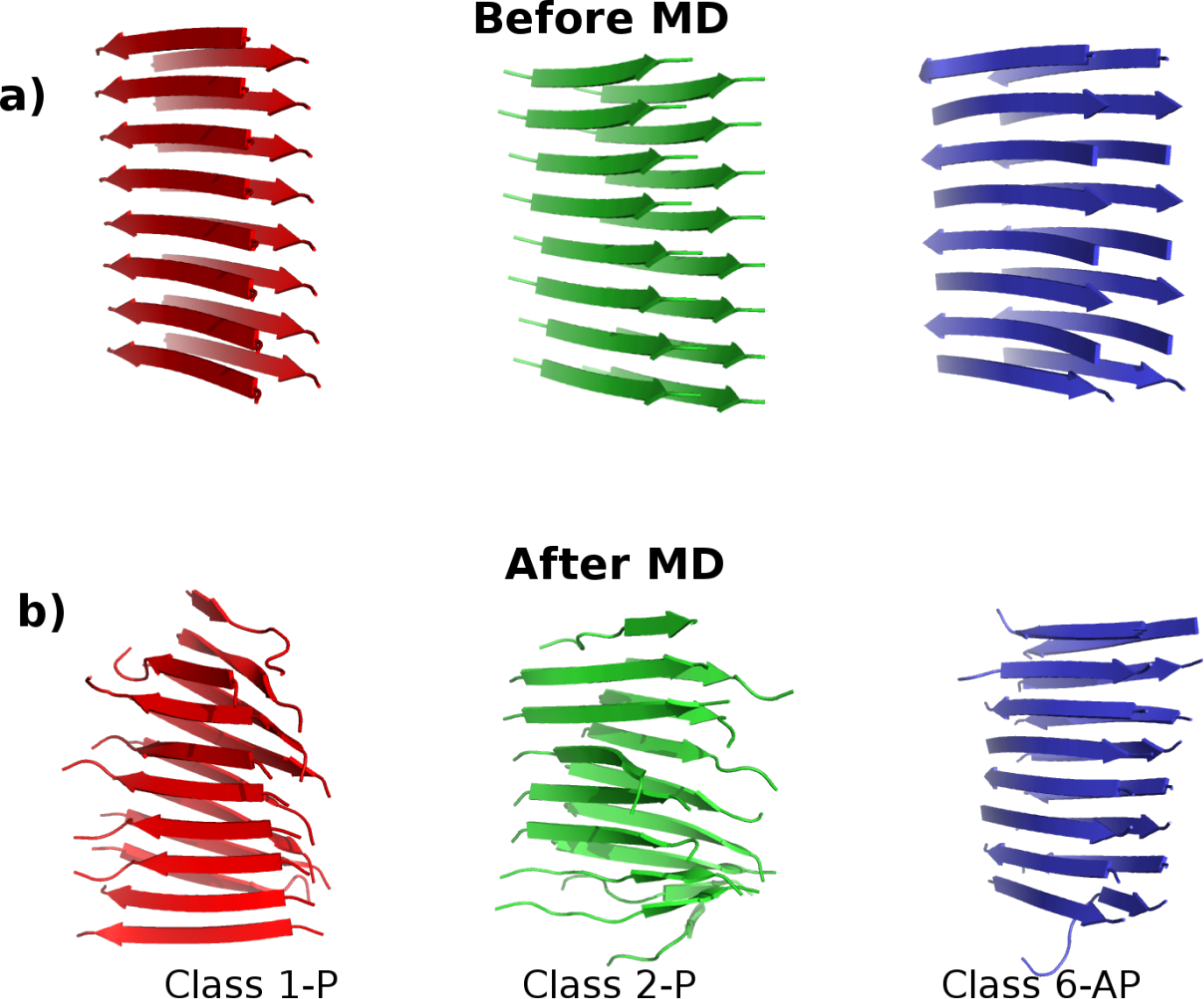
The three polymorphs of SNNFGAILSS sequence fibrils in the Class1-P (red), Class2-P (green) and Class6-AP (blue) symmetry-packing classes. (a) Models before the molecular dynamics simulation; (b) the structural changes in each model at the conclusion of 20 ns of MD in explicit solvent (water molecules omitted for clarity).

In this work, three polymorphs of fibrils formed from 10-residue fragments of the amylin protein (sequence SNNFGAILSS) as structurally determined by ssNMR [24] are simulated in full atomistic detail using molecular dynamics (MD). These models are classified according to symmetry packing classes, after the Eisenberg steric zipper nomenclature [25–26], as Class 1 (parallel both within each β-sheet and between the pair of stacked sheets), Class 2 (parallel within each β-sheet but antiparallel between stacked β-sheets) and Class 6 (antiparallel β-sheets stacked in a parallel orientation), as shown in Figure 1. The SNNFGAILSS sequence is particularly interesting in that both parallel and antiparallel polymorphs are simultaneously observed under identical growth conditions [24]. Moreover, a separate ssNMR study only observed a single fibril type in the antiparallel configuration, possibly due to the use of different terminal capping groups [27]. Consequently, the differences in energetic and mechanical stability between polymorphs of SNNFGAILSS present a unique system to study the relevant interactions that play key roles in determining their observed properties.

We have used steered molecular dynamics (SMD) simulations to probe how the packing and steric arrangements found in the three different polymorph symmetry classes influence fibril mechanical behaviour. The fibrils are probed from different directions, using distinct pulling geometries that we developed previously to study the role of the peptide sequence in modulating amyloid mechanical properties [23]. The SMD pulling geometries are designed to disrupt the stabilising hydrophobic core and backbone hydrogen bond networks from a variety of directions. We then assess how the mechanical response in the simulations is affected by doubling the length of the model fibrils, and how the mechanical properties are modulated by incorporating chemical capping groups to neutralise the N- and C-termini of the peptides. In a previous simulation study to determine the sequence dependence of the resistance of amyloid fibrils to mechanical stress by using SMD [23], we highlighted the importance of structural defects within the model fibrils in determining their mechanical properties. Similarly, in this paper we pay particular attention to the role played by structural defects in the ability of the three different polymorphs of the 10-residue amylin fragment to resist an applied force. The calculations reveal a hierarchy of factors that govern the mechanical resilience of defect-containing fibrils subjected to forces applied in silico.

## Results and Discussion

**Figure 2:**
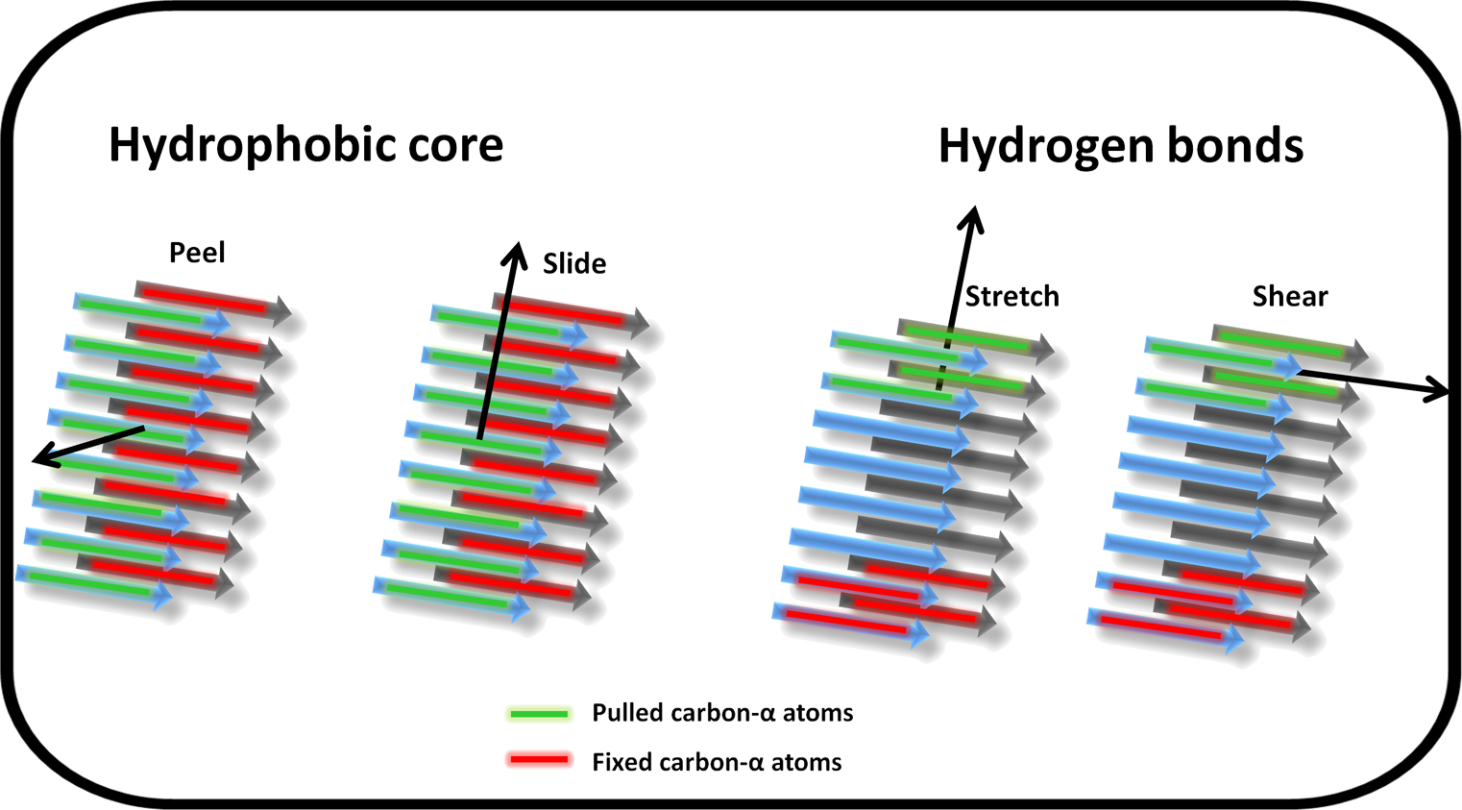
Schematic representation of the four pulling geometries used to mechanically probe the fibril models. The centre of mass of the carbon-α atoms in the peptide marked in green are pulled in the direction shown by the arrow at a constant velocity whilst the carbon-α atoms in the peptide marked in red are fixed for the duration of the simulation. All other atom types are free to move unrestrained. “Peel” and “slide” simulations probe the hydrophobic core interactions while “stretch” and “shear” interrogate the hydrogen bond networks parallel and perpendicular to the fibril long axis respectively.

To characterise the mechanical response of the fibril polymorphs, following 20 ns of standard MD to equilibrate the fibrils models, SMD simulations were carried out using the pulling geometries shown schematically in Figure 2 to probe the fibrils from different directions. Each pulling-mode simulation was repeated four times, and the mechanical properties were characterised by the average peak force measured over the four independent simulations. The nomenclature adopted throughout is Class1-P (parallel β-sheets), Class2-P (parallel β-sheets) and Class6-AP (antiparallel β-sheets).

### Mechanical responses of 8 × 2 fibril models

**Figure 3:**
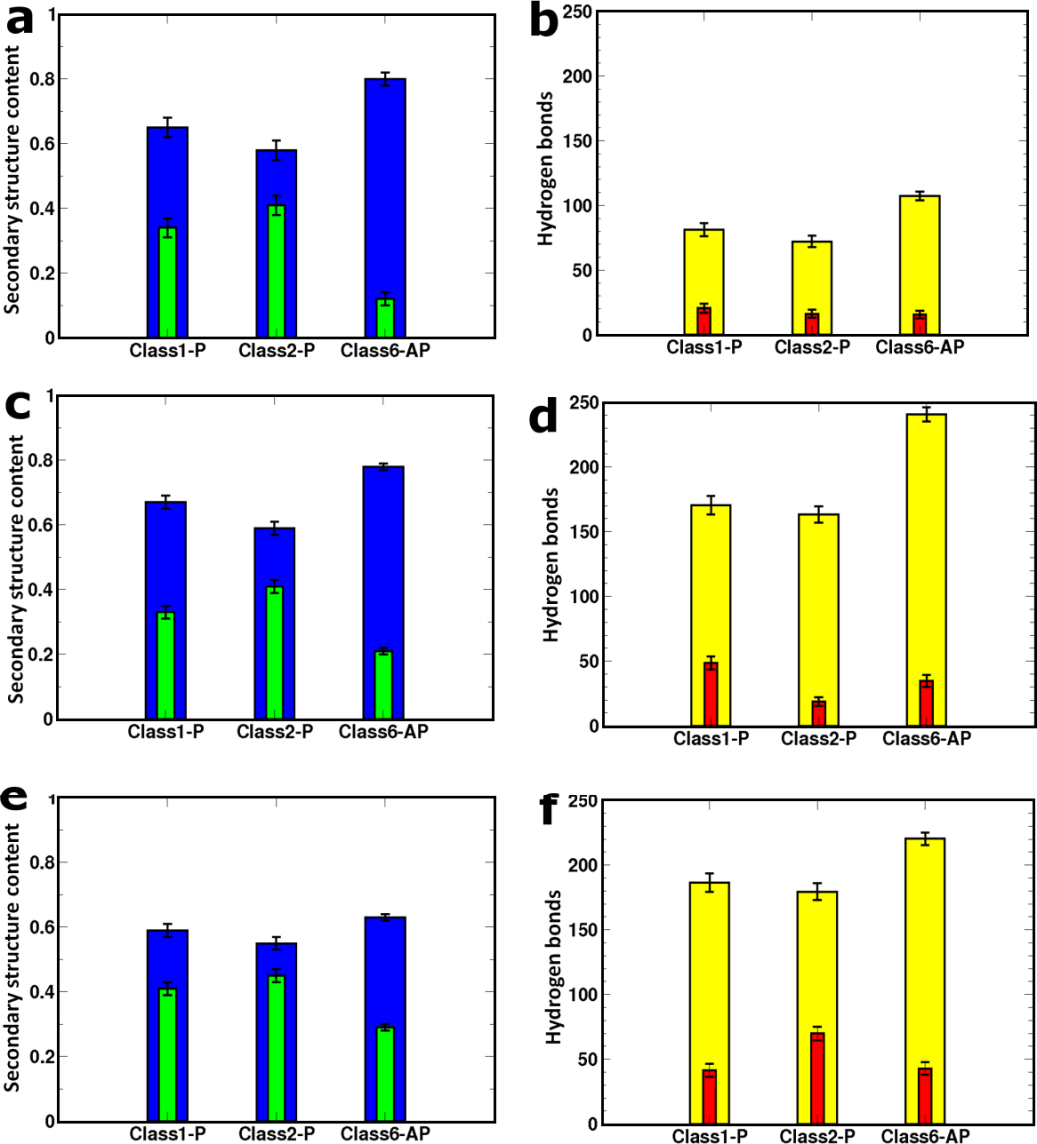
Left and right columns show the mean fraction of secondary structure content and hydrogen-bond numbers, respectively. The details for the 8 × 2 fibril models are in panels (a) and (b). On the left, blue bars show β-strand content and green bars show random coil conformations. On the right, the mean number of interstrand backbone (yellow) and side-chain (red) hydrogen bonds are shown. Panels (c) and (d) relate to the free-terminal-ended 16 × 2 models while panels (e) and (f) are for the capped 16 × 2 fibrils. The secondary structure content and hydrogen-bond analysis is computed from the final 10 ns of MD.

**Figure 4:**
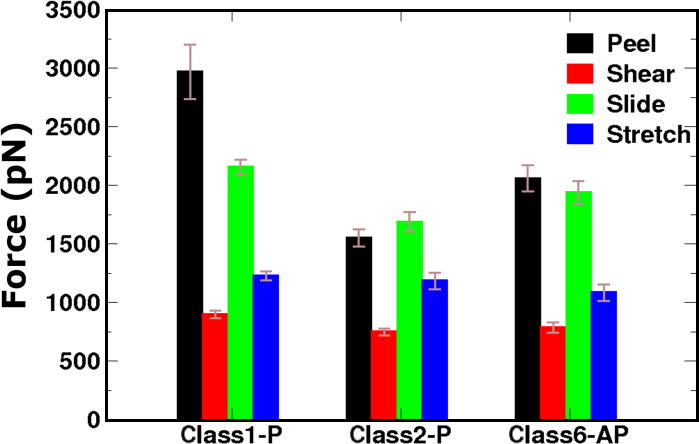
Mean peak force for the three fibril polymorphs (8 × 2 models) obtained from four repeat simulations of each pulling mode with error bars showing the standard error in the mean.

**Figure 5:**
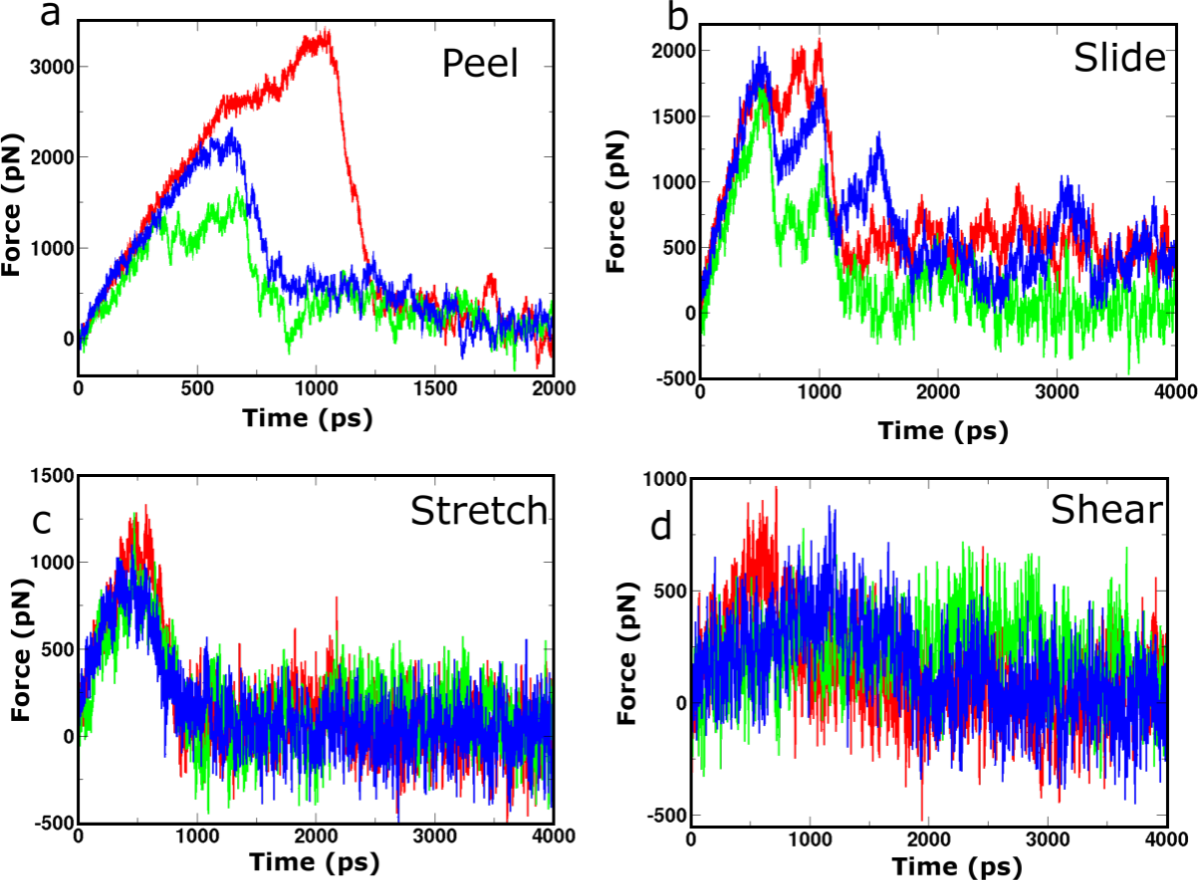
Selected force profiles recorded during SMD simulations for the class 1-P (red), class 2-P (green) and class 6-AP (blue) 8 × 2 fibril polymorphs. Panels (a), (b), (c) and (d) show the profiles from which the highest peak force was measured for each polymorph during probing by “peel”, “slide”, “stretch” and “shear” SMD pulling modes, respectively.

We first model the fibril polymorphs as two interfaced β-sheets, each of which comprises eight peptides (8 × 2 models), as shown in Figure 1. Figure 3a shows that all of the models contain some degree of structural disorder after the 20 ns of standard MD (used to equilibrate the fibrils prior to SMD), with the most ordered structure (Class6-AP) containing 80% β-sheet content, and the most disordered (Class2-P) containing only 58%. We subjected each of the three polymorphs to the four pulling modes in Figure 2, and recorded the peak forces exerted (as shown in Figure 4). Force profiles from which the highest peak forces are measured for each polymorph during the four different SMD pulling modes are shown in Figure 5. All three fibril polymorphs demonstrate an anisotropic response to mechanical probing. Similar mean peak forces are required to break the fibrils when the hydrogen-bond networks are probed (“shear” and “stretch”). There are however, very distinct responses in the SMD simulations that probe the hydrophobic core interactions (“peel” and “slide”).

**Hydrophobic core disruption:** The largest mean peak forces for both hydrophobic core probing modes (“peel” and “slide”) were recorded for the Class1-P polymorph. The molecular basis behind the relative ranking in mean peak force between the polymorphs can be understood by examining the intersheet interfaces that are affected during the SMD simulation. Both “slide” and “peel” modes disrupt the electrostatic interactions between the charged termini and force the hydrophobic core to be exposed to solvent molecules. For the three polymorphs containing eight peptides in each of the two stacked β-sheets, we observe a correlation between the mean peak force and the intersheet electrostatic interaction energies (Table 1) that arise due to the unique packing arrangements of the monomer β-strands. The Class1-P polymorph has the most favourable electrostatic energy between the stacked β-sheets because these are arranged in an antiparallel configuration, which brings the oppositely charged C- and N-termini close together. However, since Class2-P is in a parallel arrangement both within an individual β-sheet and within the stacked pair, this polymorph has the least favourable electrostatic interaction between the sheets of the three. The fact that the Class 6 polymorph is comprised of antiparallel β-sheets stacked in a parallel configuration places it intermediate between the other two. The correlation between the peak force and the electrostatic interfacial energy demonstrates how the details imposed by polymorphic arrangements of the peptides in the fibril can determine the mechanical characteristics when a force is applied in a particular direction.

**Table 1:** Thermodynamic properties of 8 × 2 fibrils.^a^

Model	Δ*G*_electro_ [kcal/mol]	Δ*G*_solv_ [kcal/mol]	Δ*G*_vdw_ [kcal/mol]	Δ*G*_Binding_ [kcal/mol]

**Class 1-P**	−2457.79 ± 4.17	2466.82 ± 4.01	−152.46 ± 0.31	−143.43 ± 0.62
**Class 2-P**	938.98 ± 1.79	−849.46 ± 1.66	−198.60 ± 0.46	−109.08 ± 0.45
**Class 6-AP**	−254.94 ± 1.05	359.97 ± 0.84	−257.77 ± 0.40	−152.74 ± 0.52

^a^The interactions of the interface between the pair of β-sheets are decomposed into electrostatic (Δ*G*_electro_), solvation (Δ*G*_solv_) and van der Waals (Δ*G*_vdw_) energy terms, which all contribute to the binding free energy (Δ*G*_Binding_). Analysis is from 1 ps snapshots of the final 10 ns of unrestrained MD as calculated by the MM-PBSA method. The mean energies are expressed in units of kcal/mol, with the standard error in the mean.

**Hydrogen-bond-network response:** In the two pulling geometries (“shear” and “stretch”) that primarily interrogate the hydrogen-bond networks, similar mean peak forces were recorded for all three polymorphs. The stretch and shear simulations probe the interpeptide hydrogen networks in directions parallel and perpendicular to the fibril axis respectively. Figure 4 shows that pulling parallel to the hydrogen bond network results in higher peak forces than when pulling across it. This implies that the hydrogen-bond network provides a cooperative resistance to the forces applied in the direction of the long axis. A surprising aspect of these simulations is that the polymorphs record virtually identical mean peak forces when subjected to “stretch”, in spite of the fact that they contain different numbers of hydrogen bonds (Figure 3b). Moreover, a systematic simulation study of the relationship between thermodynamic stability and the symmetry class of fibrils has shown that in (non-Q/N)-rich sequences, the antiparallel fibrils tend to be more energetically stable than their parallel counterparts [28], which would suggest that the Class6-AP polymorph should exhibit the most resilience to stretching forces. However, prior to SMD, the Class1-P, Class2-P, and Class6-AP structures consist of 34%, 41% and 12% random coil conformations, respectively (Figure 3a), indicating that defects are present in all fibril models. These defects can dominate the mechanical response of the fibrils in a particular pulling direction by providing weak points that are liable to fracture, as we have previously described [23].

### Mechanical response of 16 × 2 fibril models

**Figure 6:**
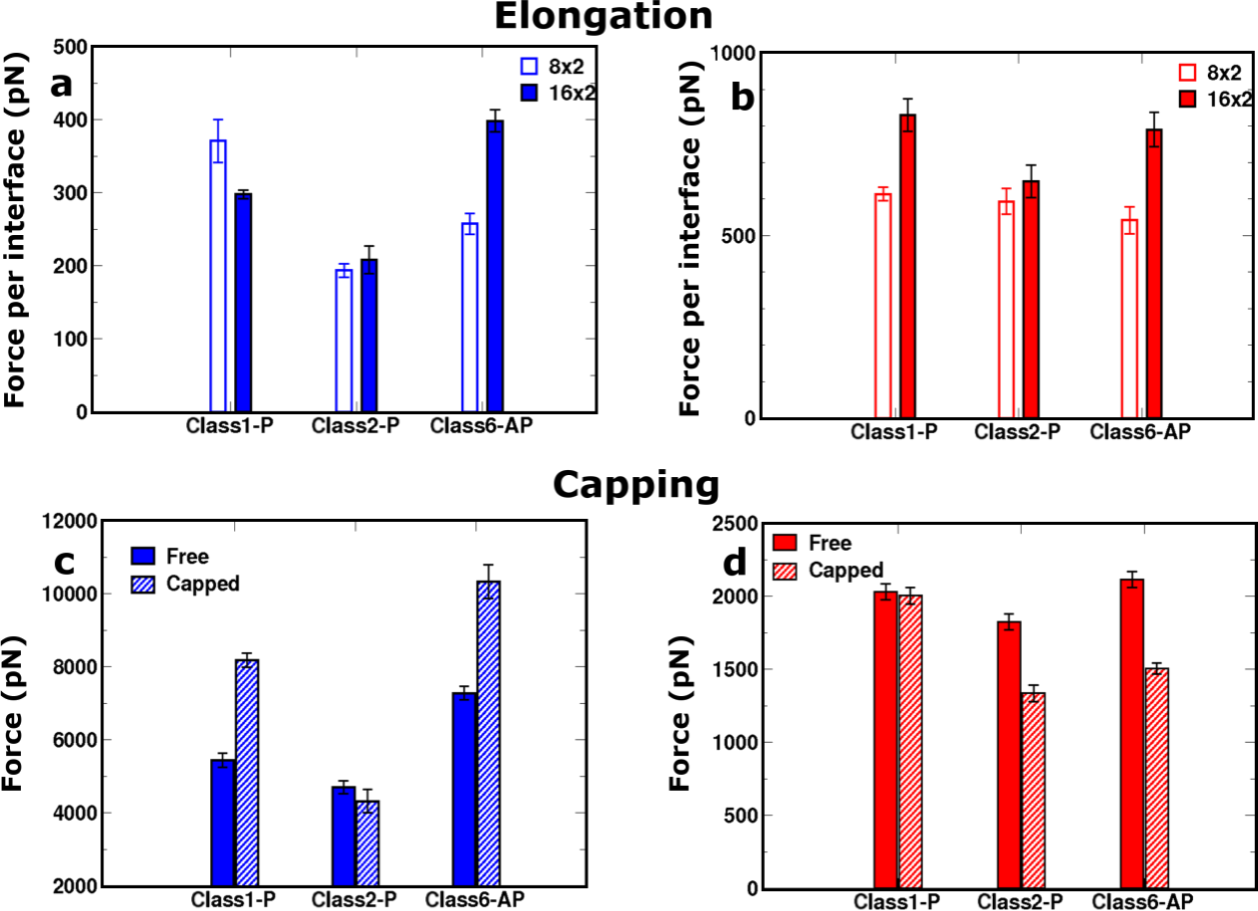
**Elongation:** The comparative response of the 16 × 2 (filled bar) and the 8 × 2 (empty bar) sized fibril models for the (a) “peel” and (b) “stretch” pulling directions. In both cases, the mean peak force is normalised by the number of interfaces interrogated during the simulation. **Capping:** The comparative response of uncapped (filled bar) and capped (striped bar) for the (c) “peel” and (d) “stretch” directions.

Having demonstrated that pulling along the long axis of the fibril by the stretch deformation mode is sensitive to the presence of structural defects within the fibrils, we then examined how the peak force changes when the fibril doubles in length from eight peptides in each of the stacked β-sheets (8 × 2 peptide arrangement) to 16 (16 × 2 fibril model), since there is a greater probability that structural defects will be present in longer fibrils. The influence of fibril length on mechanical properties has already been demonstrated by using a normal mode analysis in conjunction with a coarse-grained elastic-network model based on SNNFGAILSS fibrils [29], which showed that the bending rigidity increases up to a critical length; however, it is not possible to assess the importance of defects within such a coarse-grained model. For direct comparison between the different fibril lengths, the mean peak force during SMD was normalised to the number of interfaces probed. Figure 6b shows that all three fibril models register an increase in the mean peak force per interface upon elongation. This indicates that the increase in the total number of hydrogen bonds between the fixed and pulled ends of the fibrils results in higher peak forces being required to induce mechanical failure. This implies that there is a degree of cooperativity in the resilience of these short fibres, which arises from the increased length of the hydrogen-bonding network along the long axis of the model fibrils. However, the relative gains in the mechanical resistance appear to be unique to each polymorphic model. Doubling the length for Class1-P and Class6-AP models leads to an increase in the mean peak force per interface of 35% and 46%, respectively, whilst for Class2-P the increase is marginal at 9%. The ranking of peak force per interface amongst the polymorphs is reflected in the total number of backbone and side-chain hydrogen bonds present in each model (Figure 3d).

**Figure 7:**
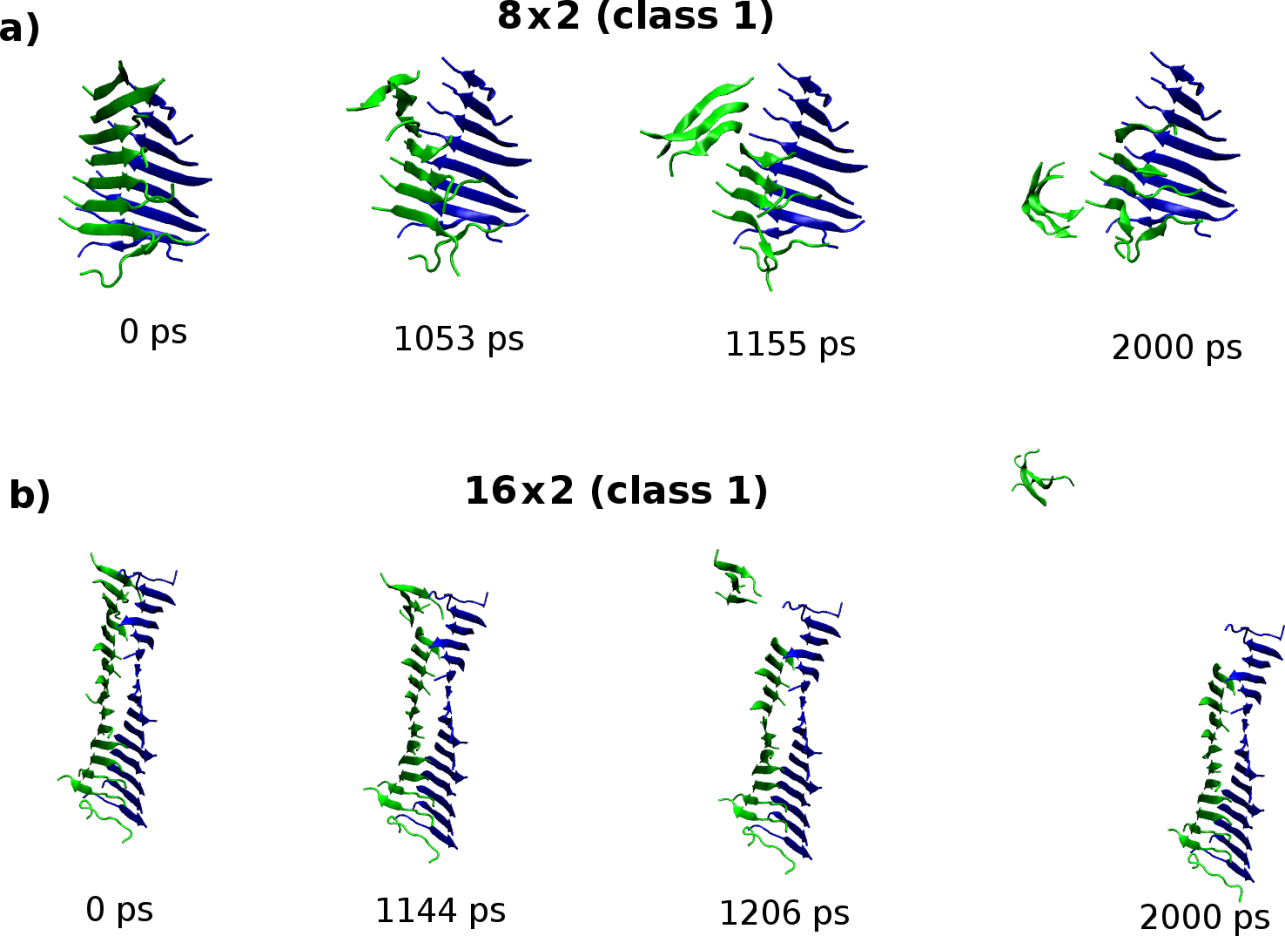
Molecular configurations sampled from the “peel” SMD trajectory for the (a) 8 × 2 and (b) 16 × 2 fibril models of the class1-P polymorph. The carbon-α atoms in the peptides coloured green are pulled while carbon-α atoms in peptides shown in blue are fixed. In contrast to the 8 × 2 fibrils, the response of the 16 × 2 fibrils leads to an exposure of only a small fraction of the hydrophobic surface.

We also investigated the dependence of the mechanical response to the “peel” deformation, which interrogates the strength of the hydrophobic interface between the pair of stacked β-sheets along the long axis of the fibril (Figure 6a). In contrast to the shorter (8 × 2) aggregates, applying peel SMD to the 16 × 2 fibrils results in a fragmentation of only a small fraction of the hydrophobic surface (Figure 7). Consequently it was not possible to relate the peak force measured to the thermodynamic stability of the hydrophobic interface, because it is not completely disrupted during the deformation. We conclude that to understand the mechanical robustness of fibrils, it is necessary to have information about the structure of the fragments that result, as well as the structure of the unperturbed fibrils themselves.

### Mechanical modulation of 16 × 2 fibril models by N- and C-terminal capping

**Figure 8:**
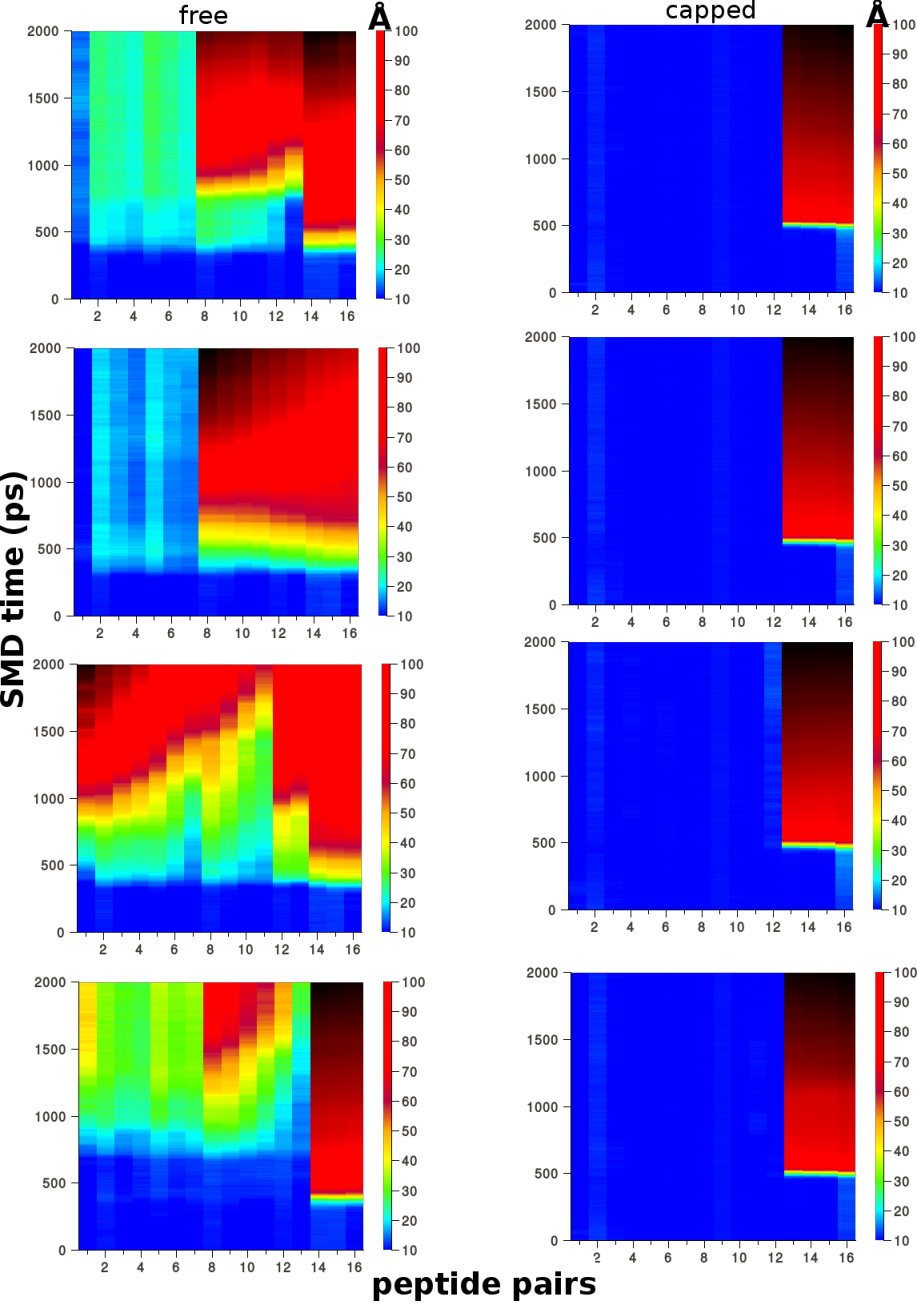
Plots showing the displacement between peptide pairs on opposite β-sheets during the “peel” SMD for the Class1-P polymorph model. The free (left) and capped terminal (right) models are each compared from four independent simulations. The colour scale is the centre-of-mass distance between pairs in angstroms (Å), the *x*-axis is the pair number (total of 16), and the *y*-axis is time in picoseconds.

We also explored how the mechanical properties of the polymorphs are affected by the addition of terminal capping groups at both ends of each peptide strand (N-terminal acetylation and C-terminal amidation), which neutralises the charged groups at both ends of the peptide monomers. The mean peak forces for fibrils of length 16 × 2 were compared for capped and uncapped models using the “peel” and “stretch” SMD modes (Figure 6c and Figure 6d). In the ”peel” pulling geometry, which gives rise to an incomplete separation of the hydrophobic interfaces for these longer fibrils, the fragmentation mechanisms between capped and uncapped models are distinct, as shown in Figure 8. Figure 8 shows a comparison of the evolution of the distances between peptide pairs on opposite β-sheets for the class1-P model during each set of repeat peel SMD simulations. The resistance mechanism for the charged-termini models shows that the separation of the peptide pairs occurs gradually, i.e., the fibrils have ductile characteristics. In stark contrast, the capped models undergo significant displacements over a very short period of time; consequently, they are more brittle than the capped counterparts. We hypothesise that this is due to the modification of the electrostatics by the addition of capping groups. In the capped case, these fibrils break suddenly because the interactions stabilising the fibrils (hydrogen bonding and hydrophobic forces) are short-ranged in comparison to the long-range electrostatic interactions within the uncapped fibrils. This highlights how a relatively simple modification at the terminus end can have a significant impact on the mechanical character of amyloid fibrils formed from short peptide sequences.

**Figure 9:**
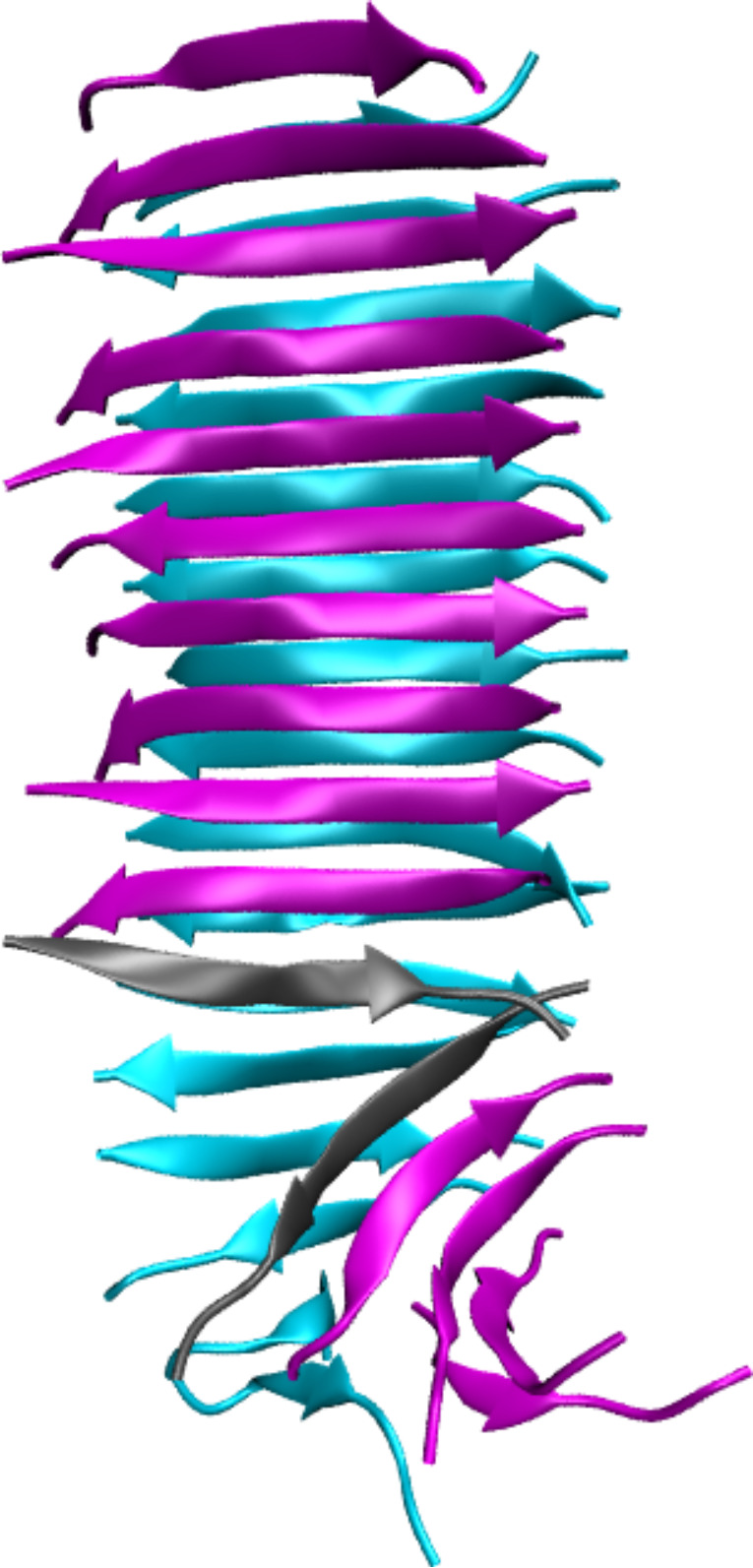
Molecular conformation of the capped Class6-AP fibril prior to SMD simulation. The misaligned peptide strands on the purple β-sheet, highlighted in grey, are significantly splayed and have reduced interpeptide hydrogen bonding. This structural defect site is the first point of failure leading to the reduced peak force for this fibril model.

The response of the capped and uncapped fibrils to SMD by the “stretch” SMD pulling mode was determined by both the number and the nature of the defects present in the fibril models. Although both the Class1-P and Class2-P polymorphs show an increase in the number of hydrogen bonds when the termini are capped, the number of ordered β-sheet secondary structures is reduced, indicating an increased number of defects within the aggregates. Consequently, the mean peak force per interface required to break the fibrils is reduced or remains the same when the termini are capped. The behaviour of the Class6-AP polymorph, which shows a large reduction in the mean peak force for the capped fibrils, provides a particularly striking example of how the response of fibrils to an applied force can be dominated by the molecular details of the defects present. Figure 9 shows the starting configuration for the SMD simulations for the capped fibril model. This polymorph developed a substantial crack defect in the one of the paired β-sheets prior to SMD, which substantially reduced the ability of this fibril model to resist “stretch” relative to its uncapped counterpart.

## Conclusion

We have performed a series of SMD simulations to mechanically probe three polymorphs of fibrils formed from the SNNFGAILSS peptide sequence. The results collectively demonstrate how the mechanical response of fibrils is directly related to the peptide packing arrangements and the number and nature of the defects present within the models. The small model fibrils investigated in this study are rich in structural defects, because they lack the stabilisation from crystal packing within a larger aggregate. Consequently, we have been able to characterise how the nature and presence of such defects influences their mechanical response. However, this precludes the investigation of effects such as crack propagation on the material properties of the model fibrils, because these occur over longer length scales. Our simulations suggest a hierarchy of factors that govern the mechanical resilience of fibrils subjected to stretching forces, which we list below in order of importance:

(1) If defects are present that are sufficiently severe that there is an absence of hydrogen-bonding interactions between one β-strand and the next within one of the paired β-sheets (e.g., the capped Class6-AP polymorph), then this defect will act as a weak point when a fibril is subjected to the “stretch” deformation, and it will rupture at lower forces than an equivalent fibril in which this defect is absent. This is illustrated by the behaviour of the capped Class6-AP polymorph, which disassociates at anomalously low forces given the number of hydrogen bonds it contains due to the presence of a crack defect in one of the paired β-sheets (as shown in Figure 9).

(2) If the fibril models contain disordered regions that have reduced hydrogen bonding interactions within a given β-sheet, but which nevertheless maintain a degree of interaction with consecutive monomers, then the fibril will be weaker when subjected to “stretch” than one with a perfectly ordered β-sheet structure. This is illustrated by the behaviour of all polymorphs subjected to the stretch deformation.

(3) If the fibrils contain a high degree of order at the interface being interrogated, and if the pulling mode applied results in a substantial separation of this interface, then the peak force required to cause mechanical failure of the fibril will be correlated with the interfacial energy. This is illustrated by the behaviour of the 8 × 2 polymorphs subjected to the “peel” deformation. We hypothesise that only for polymorphs containing a very high degree of ordered β-sheet secondary structure would the response of the fibrils to “stretch” be determined by the difference in hydrogen bonding between idealised parallel and antiparallel β-sheets.

Our simulations of amyloid polymorphs illustrate the general principles that must be considered when evaluating and comparing the mechanical properties of amyloid fibrils containing structural defects. While fibrils formed from an 11-residue fragment of transthyretin, full length and α-chain insulin fibrils and an 84-residue SH3 domain have been reported that contain extremely high degrees of structural order, with defects present in approximately one molecule in every 1000 along the fibril axis [30], experiments which have probed the mechanical properties of α-synuclein and full-length transthyretin under high-pressure conditions have shown that their robustness is indeed dominated by the presence of defects within the hydrophobic core [31]. From our understanding of the crystallisation of inorganic substances, such as minerals and ceramics, it is known that the number of defects will depend critically upon how these crystals were grown, including factors such as the rate of growth, the presence of surfaces or impurities, and whether the solution was agitated. If amyloid fibres with bespoke mechanical properties are to be used in nanotechnology, it will be necessary to assess the reproducibility of the experimental conditions used to produce the fibrils carefully, because small changes in the manufacturing could potentially alter the polymorphic form or the number density of defects present, and substantially affect mechanical robustness. Moreover, recent combined experimental and simulation studies of pore formation in membranes (which is implicated in amyloid toxicity) by oligomeric aggregates of Aβ_9–42_ have shown that the stress associated with tightly bending the fibrils to form a cylindrical channel induces large defects in previously homogeneous fibrils [32–33]. Consequently, the propensity of amyloid fibrils to form defects may also play a role in their cytotoxicity.

## Experimental

### Construction of polymorph models

The models of the parallel and antiparallel (Class1-P & Class6-AP) fibril structures were built from coordinate files determined from ssNMR [24]. The coordinate files initially consisted of a pair of β-sheets, each of which was composed of two peptides of the SNNFGAILSS sequence (2 × 2 models). These coordinates were used as templates from which longer fibrils were constructed. The Nucleic Acid Builder (NAB) software package [34] was used to make translated copies of the 2 × 2 model, which were then subsequently joined into single structures in the LEAP module of the Amber9 package [35]. The elongated copies maintained the intersheet and interpeptide separation distances found in the original ssNMR coordinates. The LEAP module also allowed for the automatic addition of hydrogen atoms (which are not resolved by ssNMR). Two fibrils sizes were constructed for each polymorph; namely a pair of β-sheets each containing eight peptides (8 × 2 model) and a pair of β-sheets each containing 16 peptides (16 × 2 model). Two versions of the 16 × 2 sized models were built with the terminal ends either free (zwitterionic form) or capped (N-terminal acetylation and C-terminal amidation). A third SNNFGAILSS model in the Class2-P symmetry configuration was also rationally designed with similar steps used to make length and capping modifications.

### Molecular dynamics simulations

Molecular dynamics simulations were run using AMBER9 [35] and NAMD2.7b1 [36] simulation packages, with the all atom AMBER99SB [37] and the CHARMM22/CMAP [38] force fields used, respectively. All models were explicitly solvated in a periodic water-box of TIP3 molecules [39] with periodic boundary conditions applied in all three directions. As the peptide sequence carried no net charge, neutralisation with counter-ions was not necessary. Long-range electrostatic interactions were calculated using the particle mesh Ewald (PME) method with a 9 Å cut-off. The models were then subjected to careful multistage equilibration with positional restraints on the solute allowing for gentle heating of each system from an initial temperature of 100 K to a target 300 K prior to MD. All bonds to hydrogen were constrained using the SHAKE algorithm allowing a 2 fs time step to be used during MD. Restraints on the hydrogen-bond distance of the interstrand backbone were then temporarily imposed for 1 ns prior to MD. All MD was run at constant temperature of 300 K using a Berendsen thermostat and constant pressure of 1 atm. The root mean square deviation (RMSD) of backbone carbon-α atoms was used to monitor convergence of the MD simulations. This was achieved within 20 and 40 ns for the 8 × 2 and 16 × 2 models, respectively.

### Steered molecular dynamics simulation

The details of the SMD protocol used are identical to those we have described elsewhere [23]; only a summary is presented here. The final configurations of the fibril models at the end of the MD were used as the starting points for SMD simulations to characterize the mechanical properties of the polymorphs. Prior to the start of SMD, each model was resolvated in a larger periodic water box in order to allow extension under force without self-interactions. The NAMD2.7b1 [36] package and Charmm22-cmap force field [38] was used to perform the simulations. The fragmentation methodology schematically shown in Figure 2 was then applied to the fibril models, with each deformation type repeated four times. The fixed/pulled atom selections only apply to carbon-α atoms in the affected peptides, with all other atom types free to move. The simulations were carried out at constant temperature (300 K) and constant pressure (1 atm). Randomised starting velocities according to the Maxwell-Boltzmann distribution in each repeat simulation were used to ensure that the trajectories sampled different areas of phase space. The SMD simulations all used a spring constant of 500 pN/Å with a constant pulling velocity of 0.01 Å/ps unless otherwise stated. The duration of the SMD simulations were 4 ns for “stretch”, “slide” and “shear” geometries, and 2 ns for the “peel” mode. The capped models required the use of different simulation parameters for error-free completion in the time step and pulling velocity (0.5 fs and 0.04 Å/ps). Thus for cross comparability, a new set of “peel” and “stretch” simulations were also run for the uncapped models with these new SMD parameters.

### Analysis methods and calculations

Secondary structure content, hydrogen bond and thermodynamic analysis of the production-phase MD simulations was performed on snapshots sampled every 1 ps from the final 10 ns of the converged trajectory. Secondary structure content was calculated with the DSSP method [40] through the PTRAJ module of AMBER 9 package [35]. The HBONDS utility in VMD [41] was used to analyse the occupancies of the backbone and side-chain interstrand hydrogen bonds. The MM-PBSA methodology as implemented in AMBER11 [35] was used to calculate the binding free energy of the intersheet interface and also the enthalpy of the fibril complex devoid of solvent molecules.
